# Reduction of Acute Rejection by Bone Marrow Mesenchymal Stem Cells during Rat Small Bowel Transplantation

**DOI:** 10.1371/journal.pone.0114528

**Published:** 2014-12-15

**Authors:** Yang Yang, Hong-Li Song, Wen Zhang, Ben-Juan Wu, Nan-Nan Fu, Wei-Ping Zheng, Chong Don, Zhong-Yang Shen

**Affiliations:** 1 Tianjin First Central Hospital, Tianjin Medical University, Tianjin, China; 2 Department of Organ Transplantation, Tianjin First Central Hospital, Tianjin, China; Université Paris Descartes, France

## Abstract

**Background:**

Bone marrow mesenchymal stem cells (BMMSCs) have shown immunosuppressive activity in transplantation. This study was designed to determine whether BMMSCs could improve outcomes of small bowel transplantation in rats.

**Methods:**

Heterotopic small bowel transplantation was performed from Brown Norway to Lewis rats, followed by infusion of BMMSCs through the superficial dorsal veins of the penis. Controls included rats infused with normal saline (allogeneic control), isogeneically transplanted rats (BN-BN) and nontransplanted animals. The animals were sacrificed after 1, 5, 7 or 10 days. Small bowel histology and apoptosis, cytokine concentrations in serum and intestinal grafts, and numbers of T regulatory (Treg) cells were assessed at each time point.

**Results:**

Acute cellular rejection occurred soon after transplantation and became aggravated over time in the allogeneic control rats, with increase in apoptosis, inflammatory response, and T helper (Th)1/Th2 and Th17/Treg-related cytokines. BMMSCs significantly attenuated acute cellular rejection, reduced apoptosis and suppressed the concentrations of interleukin (IL)-2, IL-6, IL-17, IL-23, tumor necrosis factor (TNF)-α, and interferon (IFN)-γ while upregulating IL-10 and transforming growth factor (TGF)-β expression and increasing Treg levels.

**Conclusion:**

BMMSCs improve the outcomes of allogeneic small bowel transplantation by attenuating the inflammatory response and acute cellular rejection. Treatment with BMMSCs may overcome acute cellular rejection in small bowel transplantation.

## Introduction

Small bowel transplantation (SBTx) is the sole life-saving treatment for patients with intestinal failure who fail total parenteral nutrition (TPN) or who develop irreversible liver failure due to TPN [1]. Over the last decade, the curative effects of SBTx have been improved significantly, due to advances in surgical techniques, new types of immunosuppressant and control of infection. Graft rejection rates for isolated intestinal transplants have decreased from 80% to 30%–40%, with 1-year patient and graft survival rates increasing to 90% and 80%, respectively, at experienced transplant centers [1], [2], [3]. However, long-term outcomes are not comparable with those of other organ transplants because of intense allogeneic immune response. Acute and chronic rejection, ischemia-reperfusion injury (IRI) and infectious complications remain the major problems in SBTx. Allogeneic rejection is a leading cause of graft failure or loss. Better control of acute cellular rejection (ACR) was shown to improve early graft survival and may enhance long-term survival [1], [3], [4]. Current immunosuppressive regimens have achieved better short-term outcomes. However, long-term administration of immunosuppressive agents increases the risks of unfavorable side effects, including hypertension, nephrotoxicity, neurotoxicity, post-transplant malignancy and metabolic deterioration [5]. Thus, new immunosuppressive strategies are urgently needed.

Mesenchymal stem cells (MSCs) are multipotent non-hematopoietic progenitor cells of stromal origin that have been isolated from mammalian bone marrow, cord blood and adipose tissue [6]. MSCs have regenerative, anti-inflammatory and immunomodulatory properties. These cells can regulate innate and adaptive immune responses and control the proliferation, migration and function of B, T and natural killer cells as well as inhibit immunoglobulin and antibody production, dendritic cell maturation and neutrophil activation. Some of these effects are mediated by soluble paracrine factors, such as transforming growth factor-β (TGF-β) [7], [8], [9]. Through their expression of FasL, infused MSCs can induce T cell apoptosis and promote the generation of T regulatory (Treg) cells, which may ultimately lead to immune tolerance [10], [11], [12]. Many preclinical and early clinical studies have indicated that MSCs can block the rejection of transplanted organs or even induce immune tolerance [13], [14]. These studies, however, analyzed the effects of MSCs on transplanted livers [15], kidneys [16], [17] and islet cells [18], [19], but did not assess their effects during SBTx. We previously reported that bone marrow MSCs (BMMSCs) have protective effects on rats with intestinal IRI [20], which is also a SBTx result. Now we assessed whether BMMSCs can reduce ACR after SBTx.

## Materials and Methods

### Animals

Inbred adult male Brown Norway (BN) rats (180–200 g) and male Lewis rats (200–220 g for SBTx and 100–120 g for BMMSCs) were obtained from Vital River Company (Beijing, China). Animals were housed individually in standard animal facilities, maintained on a 12-h light/dark cycle and provided with commercially available chow and tap water ad libitum for 3 days before testing. Food was withheld from both donor and recipient animals for 24 h prior to surgery. All experimental procedures were carried out in accordance with the Guide for the Care and Use of Laboratory Animals published by the National Institutes of Health (NIH publication 86–23, revised 1985), and the protocols were approved by the Animal Care and Research Committee of Tianjin First Central Hospital, Tianjin, China (Permit Number: E20130605-001A). All surgery was performed under chloral hydrate anesthesia, and all efforts were made to minimize animal suffering.

### Isolation and characterization of BMMSCs

BMMSCs were isolated from the femur and tibia of male Lewis rats (RT1^l^, 100–120 g). Red blood cells were lysed using 0.1 mol/l NH_4_Cl. The remaining cells were washed, resuspended and cultured for 4 weeks at 37°C in air containing 5% CO_2_ and in Dulbecco modified Eagle medium (DMEM)/F12 (Gibco, Carlsbad, CA, USA) containing 100 U/ml penicillin, 100 mg/ml streptomycin and 15% fetal bovine serum. The culture medium was changed every 72 h. When the third-passage cells reached 80% confluence, they were trypsinized, washed, centrifuged and resuspended at 1×10^7^ cells/ml in phosphate-buffered saline (PBS). BMMSCs were stained using antibodies against CD29, CD90, RT1A, CD45 and RT1B (Biolegend, San Diego, CA, USA) and CD34 (Santa Cruz Biotechnology, Santa Cruz, CA, USA) and analyzed by flow cytometry (FACSCalibur; Becton-Dickenson, Alaska, MN, USA). BMMSCs were also confirmed as being plastic-adherent and having a spindle-shaped morphology.

### Surgical procedures and experimental protocol

Heterotopic SBTx was performed allogeneically from BN (RT1^n^) to Lewis (RT1^l^) rats or syngeneically from BN to BN rats in a sterile field under general anesthesia using 5% chloral hydrate (10 ml/kg). The surgical techniques were similar to those previously described [21], [22]. Briefly, the entire small bowel from the ligament of Treitz to 1 cm from the ileocecal valve was removed on a vascular pedicle consisting of the superior mesenteric artery (SMA) attached to an aortic cuff and the portal vein (PV). After mobilization of the inferior vena cava and the aorta from the surrounding connective tissue, transplantation was performed by revascularization between the donor SMA and the recipient infrarenal aorta and between the donor PV and the recipient infrarenal inferior vena cava by end-to-side anastomosis. Cold ischemic time was approximately 20 min, and warm ischemic time approximately 30 min. The distal ends of the donor bowels were brought out as a stoma through the abdominal wall. Recipient rats were treated with 1×10^7^ BMMSCs as treatment group (BMMSCs) through the superficial dorsal veins of the penis immediately after the surgery, or normal saline as allogeneic controls (Allo). Isogeneic control (Iso) rats received no treatment. Nontransplanted rats served as another control (NSBTx). Five animals per group were euthanized 1, 5, 7 and 10 days after SBTx.

### Survival rate and clinical manifestations

We observed the survival rate and clinical manifestations in five recipient rats per group. Animal and graft survival rates were compared between groups using Kaplan–Meier analysis. Log-rank testing was used to ascertain the significance of differences between groups. Differences were considered statistically significant when p≤0.05. Data are presented as the mean ± SD.

### Histopathological analysis

Intestinal tissues were fixed in 10% formalin, embedded in paraffin and cut into 5-µm sections, which were stained with hematoxylin and eosin (HE). The slides were blindly reviewed and graded as described [23].

### Intestinal mucosal ultrastructure

Intestinal tissues were cut into small pieces 1 mm×2 mm and fixed in 2.5% glutaraldehyde. Ultrathin (70 nm) intestinal sections were prepared using standard techniques and examined by transmission electron microscopy (Hitachi H-600, Tokyo, Japan).

### Apoptosis assay

TUNEL (TdT-mediated dUTP-X nick end labeling) staining was performed on paraffin-embedded tissue sections using an In Situ Cell Death Detection Kit, as described by the manufacturer (Roche Biochemicals, Mannheim, Germany). As negative control, terminal deoxynucleotidyl transferase was omitted, whereas positive controls were generated by treatment with deoxyribonuclease. The slides were blindly reviewed, and the number of positive cells was counted in 10 randomly chosen fields under light microscopy (200×).

### Natural killer cell activity

Host natural killer (NK) cell function was evaluated by measuring the lactate dehydrogenase (LDH) release following co-culture with the target YAC-1 cells. Splenocytes were isolated and resuspended in 10% fetal calf serum (FCS)–RPMI 1640, and were counted and adjusted to 1×10^7^/ml. YAC-1 cells were prepared and adjusted to 1×10^5^/ml. Both cell types (100 µl each) were co-cultured in culture plates at 37°C in 5% CO_2_ for 2 h. The natural release group (100 µl YAC-1 cells+100 µl 10% FCS–RPMI 1640) and maximum release group (100 µl YAC-1 cells+100 µl 1% NP40) were used as the controls. Supernate (100 µl) was transferred from each culture dish to a new culture plate and incubated for 10 minutes at 37°C. LDH substrate (100 µl) was added to each culture plate for 10–15 minutes in the dark. The reaction was halted with 30 µl 1 mol/L citric acid. NK cell activity was calculated based on the optical density at 570 nm.

### ELISA

Recipient serum was obtained from peripheral blood, and fluid was extracted from intestinal grafts at each time point. Interleukin (IL)-10, TGF-β, IL-2, IL-6, IL-17, IL-23, tumor necrosis factor (TNF)-α, and interferon (IFN)-γ concentrations were measured by enzyme-linked immunosorbent assay (ELISA) kits as described by the manufacturer (BioSource, USA), including comparisons with standard curves. The minimum detectable concentrations were 1 pg/ml (IL-10, TGF-β, IL-2, IL-6, IL-17, IL-23, TNF-α) and 4 pg/ml (IFN-γ). The intra-coefficients of variation for all ELISAs were ≤6.

### Flow cytometry

Lymphocytes were isolated from the recipient spleens. Aliquots (1×10^6^ cells) were resuspended in 0.1 ml PBS and incubated with antibodies against CD4, CD25, and Foxp3 (eBioscience, San Diego, USA). Anti-CD4 antibody was conjugated with fluorescein isothiocyanate (FITC); the isotype antibody was mouse immunoglobulin G (IgG)2a K-FITC. Anti-CD25 antibody was conjugated with phycoerythrin (PE); the isotype antibody was mouse IgG1 K-PE. Anti-Foxp3 antibody was conjugated with PerCP-Cyanine5.5; the isotype antibody was rat IgG2a K–PerCP-Cyanine5.5. Isotype-matched control served as a control. The cells were analyzed by flow cytometry (FACSCalibur; Becton-Dickenson).

### Tregs depletion

Recipients were administered with 1×10^7^ BMMSCs and 300 µg anti-CD25 antibody after SBTx. The recipients were sacrificed after 7 days, and the graft and serum were collected for pathology, apoptosis detection, and cytokine testing.

### Statistical analysis

Results are expressed as mean ± standard deviation (SD) and compared by one-way analysis of variance. All statistical analyses were performed using SPSS Statistical Software, v. 17.0 (SPSS GmbH, Munich, Germany), with p≤0.05 considered statistically significant.

## Results

### Culture of BMMSCs

**Figure 1:**
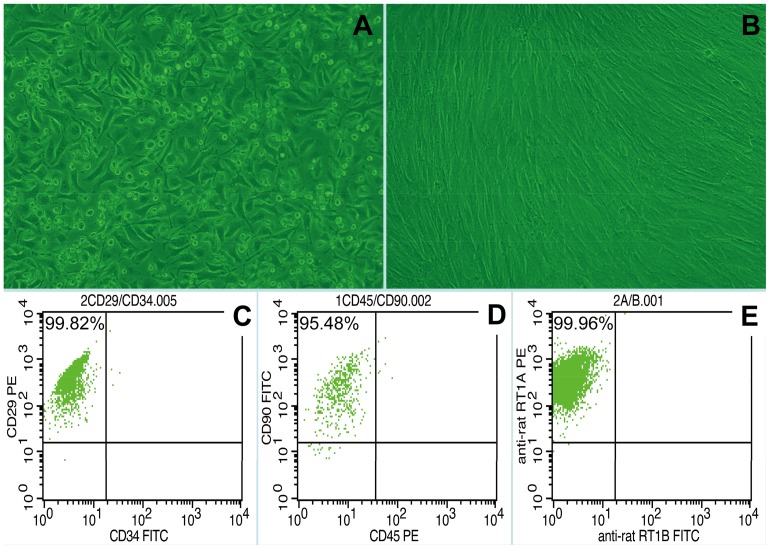
Morphology of BMMSCs in vitro and Flow cytometric analysis of third-passage BMMSCs: Morphology of first-passage (A) and third-passage (B) BMMSCs. (C) The proportion of CD29-positive and CD34-negative cells was >99%. (D) The proportion of CD90-positive and CD45-negative cells was approximately 96%. (E) The proportion of RT1A-positive and RT1B-negative cells was >99%.

The cells isolated from rat bone marrow were confirmed as being BMMSCs based on their spindle-shaped morphology, adherence to plastic and ability to differentiate into chondrocytes, adipocytes, osteocytes, and hepatocytes in vitro (data not shown). Flow cytometry showed that the BMMSCs preparations were >95% pure, and that >98% of these cells were positive for CD29, CD90 and RT1A and negative for CD34, CD45 and RT1B (Fig. 1). In agreement with previous findings [24], the percentages of CD90^+^ and CD45^−^ cells increased from 80% to >98% over the first three passages.

### Improvement of clinical manifestations and recipient and graft survival rates

**Figure 2:**
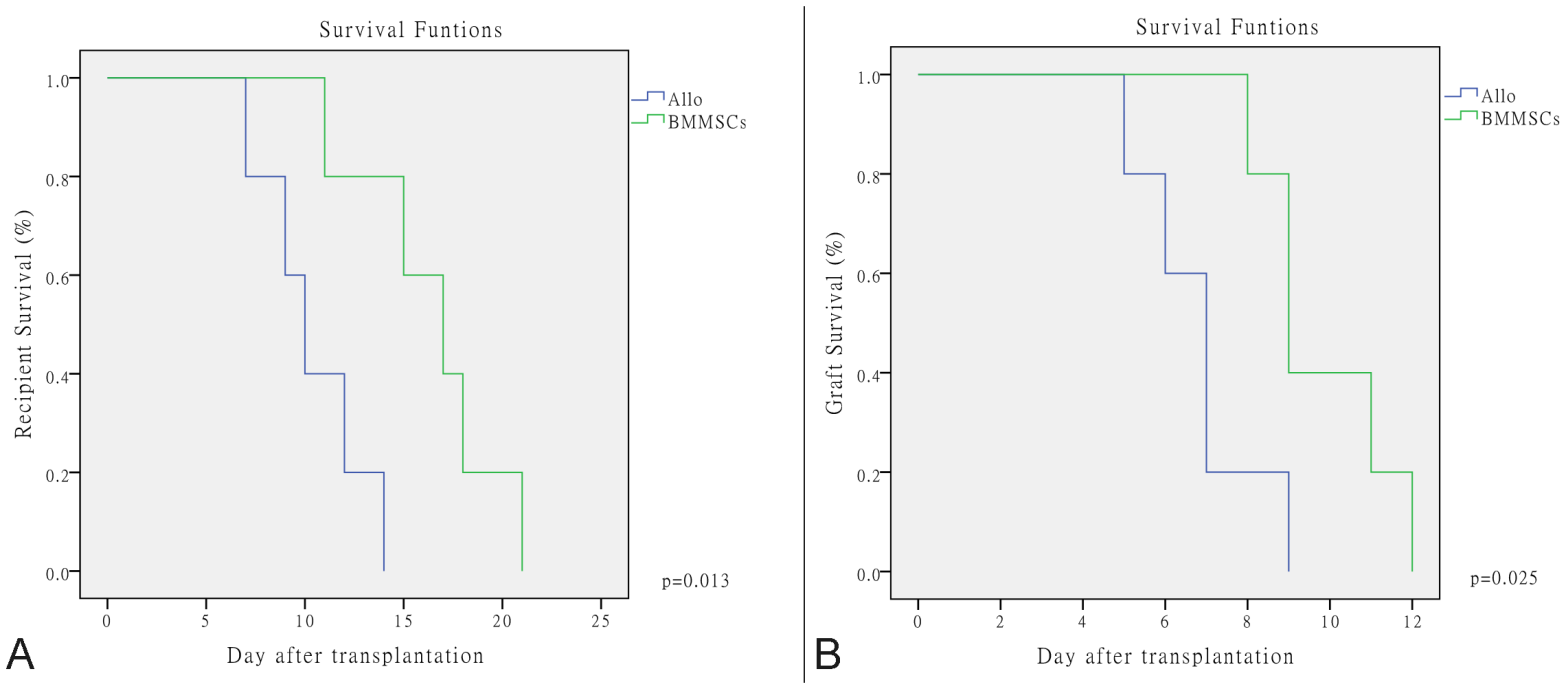
Kaplan–Meier recipient and graft survival curves. (A) Recipient survival curve. BMMSCs improved the recipient survival rate significantly (p = 0.013). (B) Graft survival curve. BMMSCs improved the graft survival rate significantly (p = 0.025).

The recipients all revived and resumed normal activity soon after SBTx. Recipients in the Allogeneic control group began to appear scruffy and their fur began to appear dull, and there were increased oral or ocular abnormal secreta 2 days after SBTx. From day 5 onwards, the stoma mucosa became red and swollen, and the recipients began to lose weight and became unresponsive, with different degrees of somnolence, asitia, and loose stools; all died within 15 days. Recipients in the BMMSC group were more active and responded more quickly to stimulation, and most survived for more than 15 days. The median recipient and graft survival times were 10 vs. 17 days and 7 vs. 9 days, respectively, in the Allo and BMMSC groups. The BMMSCs significantly improved the recipient and graft survival rates (Fig. 2).

### Histopathological analysis and grading of acute rejection in small bowel graft

**Figure 3:**
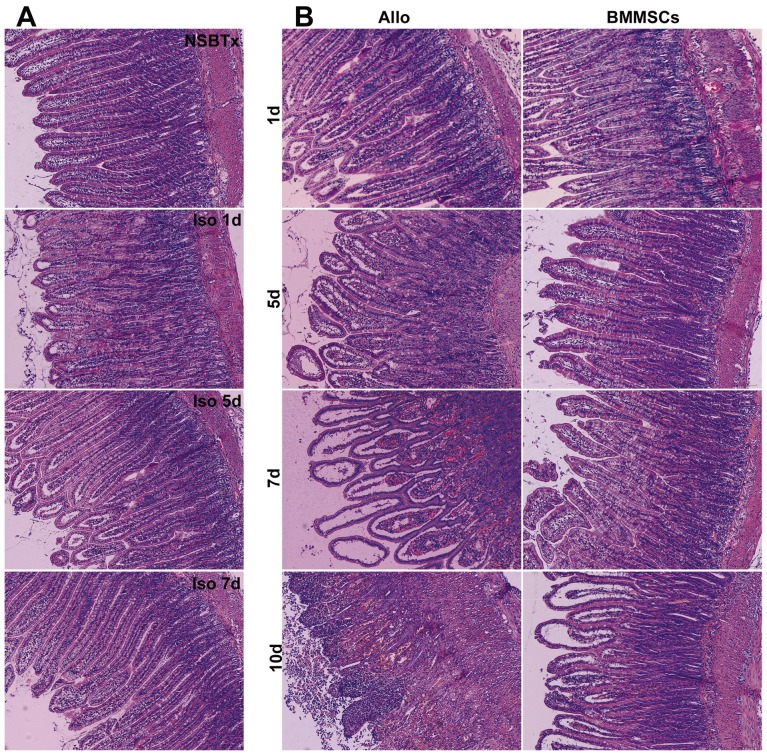
Histological sections of the rat small bowel and grading of acute cellular rejection after SBTx. (A) Nontransplanted BN rats with normal histopathological structure and crypt architecture, and isogeneically transplanted animals showing minimal cellular infiltration but no apoptotic cells or crypt destruction in the mucosa as residues of IRI on days 1 and 5, followed by repair of IRI-associated histological changes on day 7. (B) Allogeneically transplanted animals showing different degrees of rejection at each time point. ACR grading on day 7 was moderate to severe, with extensive mucosal destruction including massive damage to crypt architecture, loss of villi and heavy infiltration by neutrophils and lymphocytes. ACR was more alleviated in BMMSC-treated animals, with more integrated mucosal architecture, fewer inflammatory infiltrates and crypt epithelial apoptosis comparable to the allogeneic transplanted group, especially on days 5 and 7 (H&E staining, ×100).

**Figure 4:**
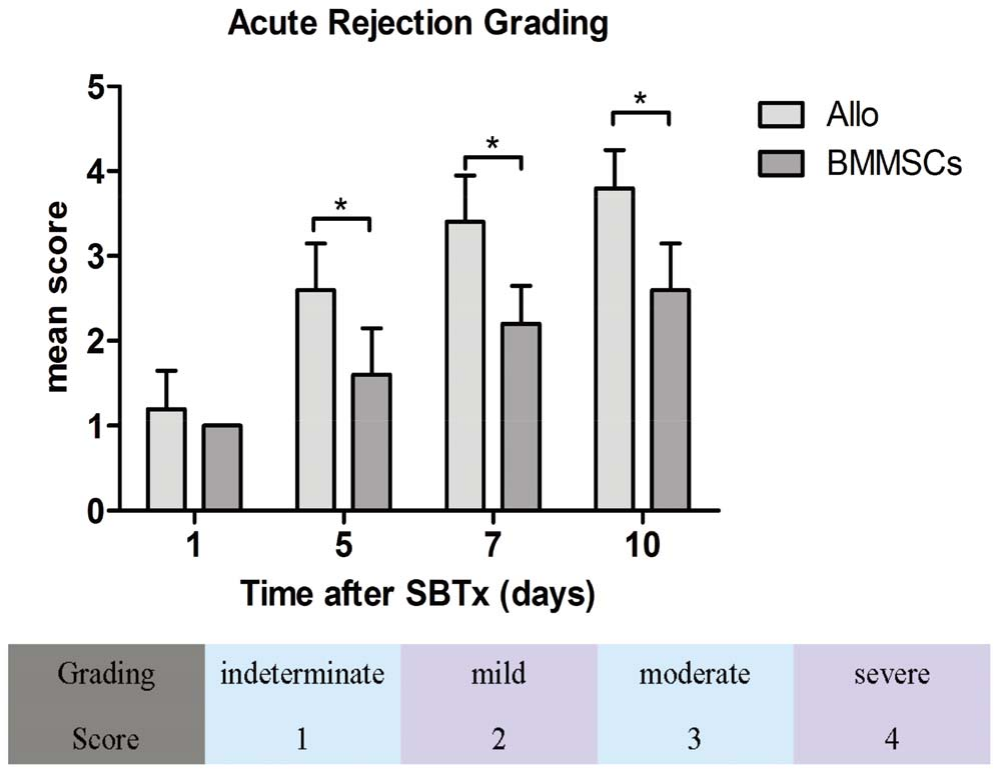
Histogram of acute rejection grading. Histogram showing that the mean histological grading scores are decreased in the BMMSC group compared to the Allogeneic control group. Data are expressed as mean ± SD (*p≤0.05).

To analyze the effects of BMMSCs on ACR of whole small bowel grafts, we assessed the histopathological grade of ACR at each time point after SBTx using a system that grades ACR as indeterminate, mild, moderate or severe, depending on findings in the mucosa and submucosa, including cell infiltration, crypt epithelial injury, apoptotic bodies in crypts, architectural distortion and mucosal ulceration [23]. Isogeneically transplanted animals showed minimal cellular infiltration into the muscle layer and mucosa following IRI, but no apoptotic cells or crypt destruction (Fig. 3A). Allogeneically transplanted animals treated with normal saline showed gradual increases in acute rejection, with moderate or severe acute rejection observed on day 7. Allogeneically transplanted animals treated with BMMSCs showed improvements after transplantation, with grade of rejection significantly lower on days 5, 7 and 10 compared with the allogeneically transplanted, saline-treated group (Fig. 3B and Fig. 4).

### Ultrastructural changes in the intestinal mucosa

**Figure 5:**
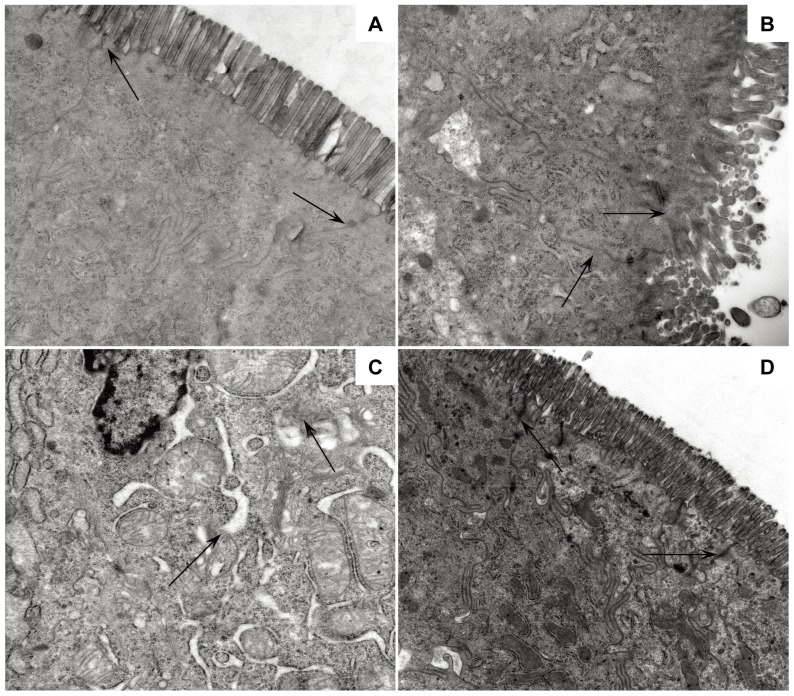
Ultrastructural pathological damage in rat intestines on day 5 after SBTx. (A) Nontransplanted rats, showing intact epithelial cells and tight junctions (TJ; arrows); ×20,000. (B) Allogeneic control rats, showing some loose microvilli, disruption of TJs (arrows) and organelles swollen due to reduced electron density; ×25,000. (C) Isogeneic control rats, showing swelling and shrinkage of epithelial cells, abnormal microvilli and organelles and disruption of TJs (arrows); ×25,000. (D) BMMSC-treated rats, showing that the microvilli and mitochondria of endothelial cells were almost normal and TJs (arrows) were not disrupted; ×20,000.

Ultrastructural changes occurring during ACR in SBTx are evident before gross or histological changes [25], with ultrastructural microvillous changes being markers of rejection in SBTx [26]. Morphological alterations of tight junctions in allograft mucosa were thought to be related to rejection after SBTx [27]. Serious disruptions in ultrastructure, including blunted, distorted and sparse microvilli, loosened adherent junctions and reduced mitochondrial matrix, were observed in both the isogeneic and allogeneic control groups on days 1 and 5. Beginning on day 7, these changes normalized in the isogeneic control group, but continued in the allogeneic control group. In the BMMSC-treated group, the microvilli and tight junctions remained normal for 7 days, with definite ultrastructural changes not observed until day 10 (Fig. 5).

### Evaluation of apoptosis

**Figure 6:**
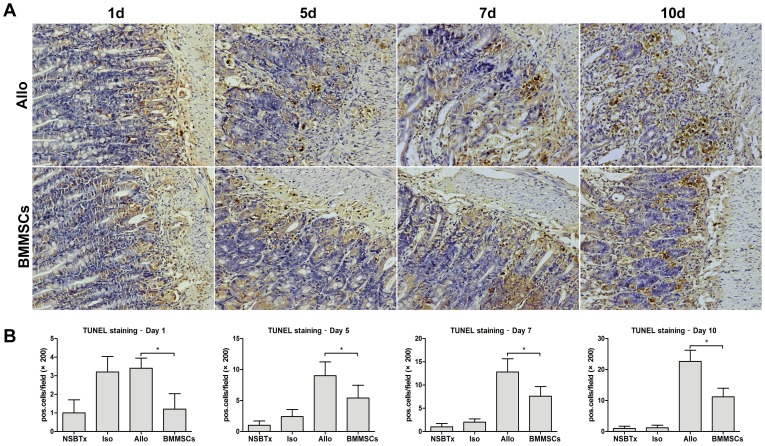
Apoptotic cells in graft mucosae. (A) Histological sections from Allogeneic control group rats and BMMSC group rats at each time point were subjected to TUNEL staining (×200). (B) Histogram showing the percentage of positive apoptotic signals in each group on days 1, 5, 7 and 10. Apoptotic cells in graft mucosae were increased significantly in the allogeneic control group, an increase significantly inhibited by BMMSC treatment (*p≤0.01).

Acute rejection is associated with increased apoptosis in the graft [28]. Using TUNEL staining, we evaluated apoptotic cells in graft mucosa after SBTx (Fig. 6A). The numbers of apoptotic cells in the intestinal mucosa were higher in isogeneic control group than in nontransplanted controls on day 1. The number of TUNEL-positive apoptotic cells was significantly higher in the allogeneic than in the isogeneic control group except day 1. BMMSC treatment significantly reduced TUNEL-positive apoptotic cells in the graft mucosa at each time point (Fig. 6B).

### Changes in NK cell activity

**Figure 7:**
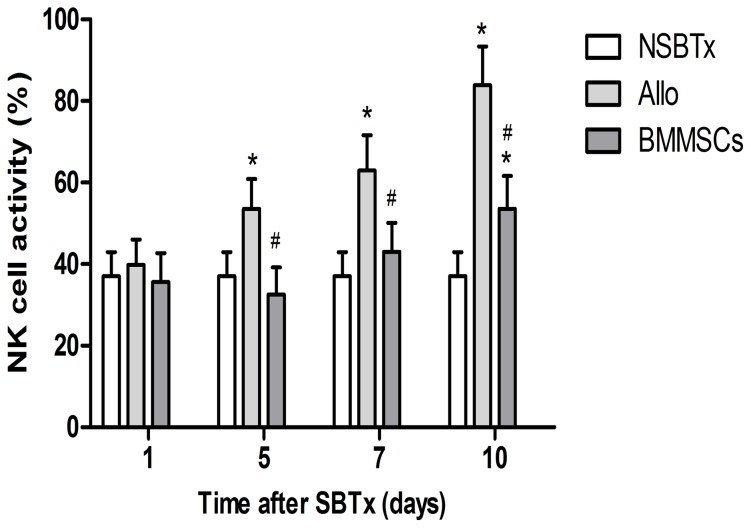
NK cell activity evaluated by measurement of LDH release in mixed culture with YAC-1 cells. NK cell activity increased rapidly in the Allogeneic control group and was significantly higher than that of the NSBTx group on day 5, 7, and 10. BMMSCs significantly reduced NK cell activity compared to the Allogeneic control group. Data are expressed as mean ± SD (p≤0.05 compared with *nontransplanted controls or ^#^allogeneic controls).

NK cell activity increased rapidly in the Allogeneic control group and was significantly higher than that in the NSBTx group on day 5, 7, and 10. There was no remarkable difference between the NSBTx and BMMSC group except on day 10, when NK cell activity in the latter increased. Furthermore, the BMMSCs significantly reduced NK cell activity compared to the Allogeneic control group (day 5, 32.51±6.70 vs. 53.54±7.30; day 7, 43.00±7.11 vs. 62.98±8.68; day 10, 53.51±8.08 vs. 83.87±9.47) (Fig. 7).

### Recipient serum cytokine concentrations

**Figure 8:**
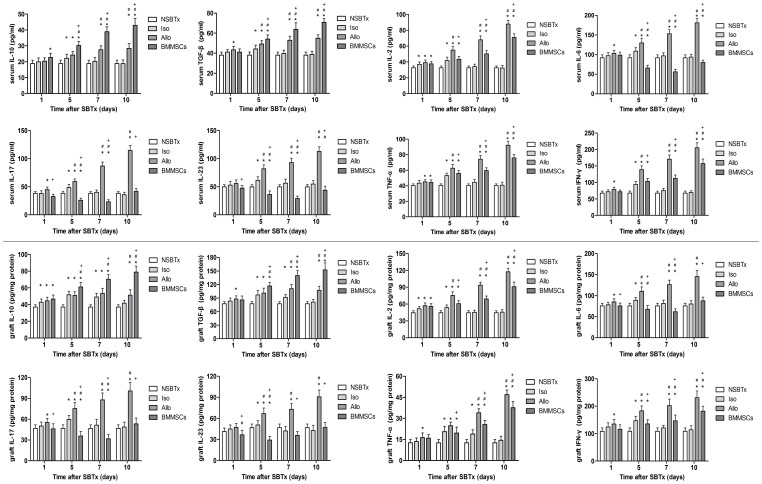
IL-10, TGF-β, IL-2, IL-6, IL-17, IL-23, TNF-α, and IFN-γ concentrations in recipient serum and intestinal grafts (S1 and S2 Tables). Cytokine concentrations in the recipient serum and intestinal grafts were measured by ELISA on day 1, 5, 7, and 10 after transplantation. Data are expressed as mean ± SD (p≤0.05 compared with *nontransplanted controls; #isogeneic controls; +allogeneic controls).

The serum concentrations of inflammatory response–related cytokines and Th1/Th2 and Th17/Treg were assayed with ELISA at the same time points after SBTx (Fig. 8). Compared with concentrations in the NSBTx group, IL-2, IL-6, IL-10, IL-17, IL-23, TGF-β, TNF-α, and IFN-γ concentrations all increased in the Isogeneic control group, peaking on day 5 and declining to normal on day 7. In the Allogeneic control group, the cytokine levels increased steadily after SBTx and were significantly higher than that in the Isogeneic control group (p≤0.05 on day 7 and 10 for IL-10; p≤0.05 on day 5, 7, and 10 for IL-2, IL-6, IL-17, IL-23, TGF-β, TNF-α, IFN-γ). The increases in IL-2, TNF-α, and IFN-γ levels were smaller in the BMMSC group and were much lower than that in the Allogeneic control group (p≤0.05 on day 5, 7, and 10 for IL-2, TNF-α, and IFN-γ). However, there was a sharper increase in their concentrations on day 10 than at the other time points. We observed downward trends for serum IL-6, IL-17, and IL-23 concentrations in the BMMSC group within 7 days, and their concentrations were significantly decreased compared to the Allogeneic control group (p≤0.05 on day 5, 7, and 10 for IL-6; p≤0.05 at each time point for IL-17 and IL-23). Serum IL-10 and TGF-β concentrations in the BMMSC group also increased rapidly and peaked on day 10, but were significantly higher than that in the Allogeneic control group at each time point (p≤0.05 on day 5, 7, and 10) (Fig. 8).

### Recipient intestinal graft cytokine concentrations

Parallel to changes in the recipient serum, IL-2, IL-6, IL-17, IL-23, TNF-α, and IFN-γ concentrations were significantly lower in the intestinal grafts of BMMSC-treated rats compared to the Allo rats on day 5, 7, and 10. The intestinal graft concentrations of IL-10 and TGF-β in the BMMSC group were significantly higher than that in the Allogeneic control group on day 5, 7, and 10 (p≤0.05 on day 5, 7, and 10 for IL-2, IL-10, TGF-β, TNF-α, IFN-γ; p≤0.05 at each time point for IL-6, IL-17, IL-23) (Fig. 8).

### Recipient spleen Treg levels

**Figure 9:**
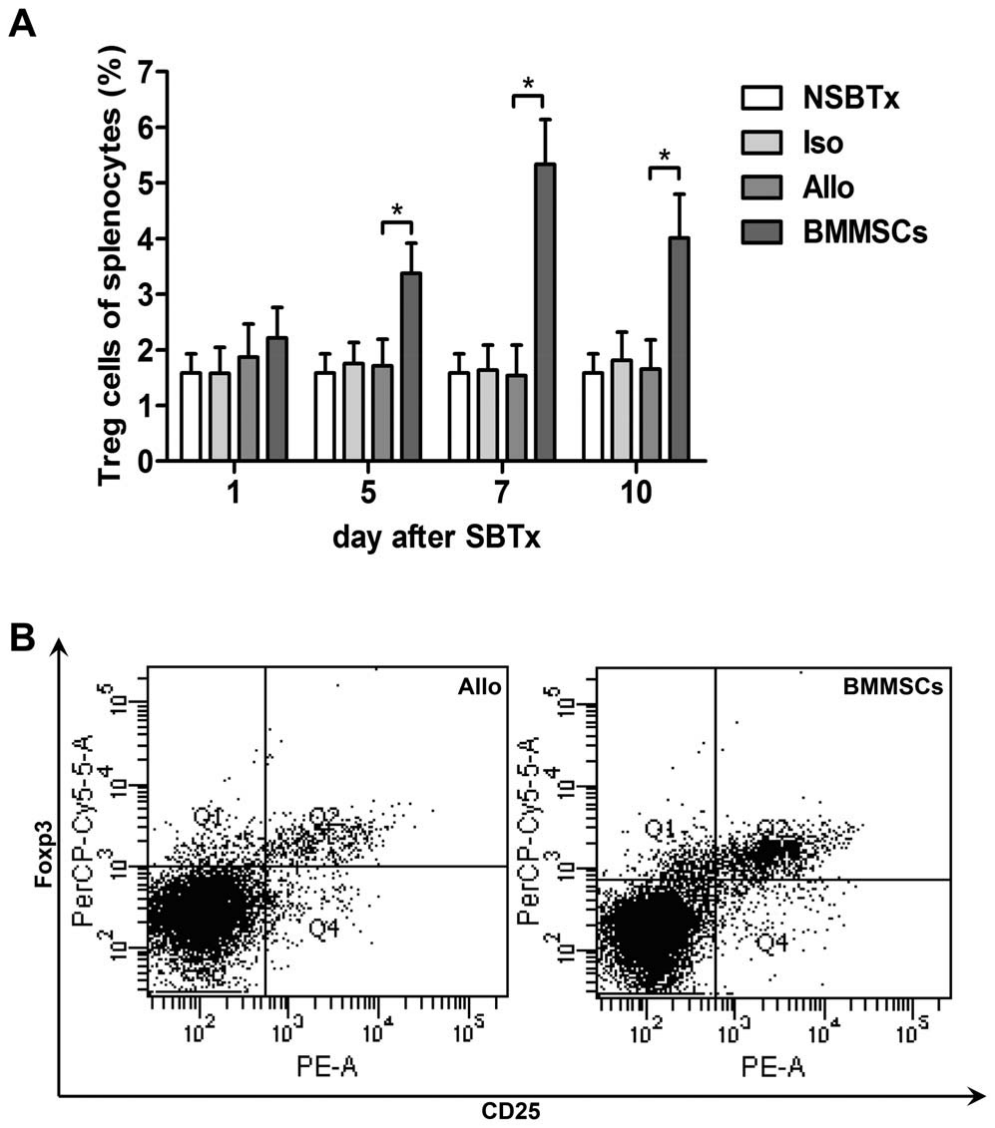
Expression of Treg cells in recipient spleen. The percentages of CD4^+^CD25^+^Foxp3^+^ cells in recipient spleens were measured by flow cytometry. (A) Scatter plot on day 7, showing that the percentage of Tregs was higher in BMMSC-treated rats than in the Allogeneic control group. (B) Histogram showing the percentages of Treg cells on days 1, 5, 7 and 10. There were no differences in isogeneic and allogeneic controls compared with nontransplanted animals, whereas BMMSC treatment significantly increased the percentages of Treg cells, especially on day 7 (*p≤0.05).

The numbers of CD4^+^CD25^+^Foxp3^+^ Treg cells in the spleens of nontransplanted, isogeneic control, and allogeneic control rats were similar at each time point. In contrast, the numbers of Treg cells were significantly higher in the BMMSC-treated group than in the allogeneic control group, especially on days 5, 7 and 10 (p≤0.01) (Fig. 9).

### Effect of Tregs on BMMSC immunomodulatory activity

**Figure 10:**
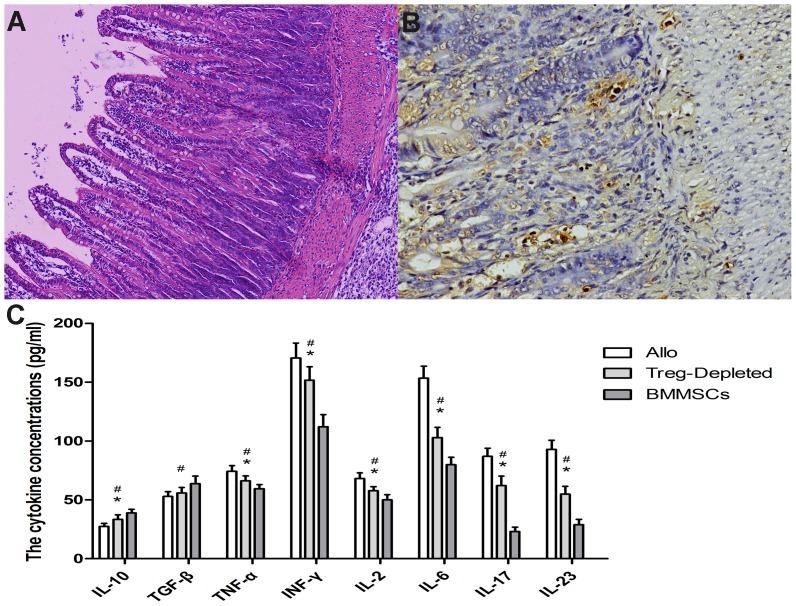
Histopathological, apoptotic, and cytokine changes in Tregs-Depleted group on day 7. (A) Histological sections showing aggravated intestinal damage induced by acute rejection, but the change was better than that in the Allogeneic control group. (B) TUNEL staining depicting the number of apoptotic cells in the Tregs-Depleted group. (C) IL-10, TGF-β, IL-2, IL-6, IL-17, IL-23, TNF-α, and IFN-γ concentrations in recipient serum. Data are expressed as the mean ± SD (p≤0.05 compared with *nontransplanted controls or ^#^allogeneic controls).

We depleted CD4^+^CD25^+^Foxp3^+^ T cells with anti-CD25 antibody to confirm the relationship between Tregs and the immunosuppressive effect induced by the BMMSCs. HE staining revealed severe intestinal damage induced by acute rejection in the Tregs-Depleted group (Fig. 10A). The degrees of rejection and apoptosis were increased and serum IL-10 and TGF-β levels were decreased; serum IL-2, IL-6, IL-17, IL-23, TNF-α, and IFN-γ levels in the Tregs-Depleted group were increased by varying degrees compared to the BMMSC group (Fig. 10B and 10C). However, there was visible improvement in the histopathological results and apoptosis in the Tregs-Depleted group compared to the Allogeneic control group, where serum IL-10 increased while serum IL-2, IL-6, IL-17, IL-23, TNF-α, and IFN-γ decreased compared to the Allogeneic control group (Fig. 10C).

## Discussion

Although improvements in immunosuppressants have resulted in better control of rejection after SBTx, the incidence of rejection remains high, with rates of acute and chronic rejection after SBTx of 90% and 30%–50%, respectively [29]. More effective immunosuppressants with fewer side effects are urgently needed to improve the outcome of SBTx. Our study was designed to investigate the role of immunoregulatory BMMSCs in suppressing ACR after SBTx.

Heterotopic SBTx was established in a major histocompatibility complex (MHC)-disparate rat strain combination (Brown Norway to Lewis), and acute rejection was assessed histopathologically and ultrastructurally. Histopathological analysis showed that acute rejection gradually increased in the allogeneic control group, with acute rejection usually occurring on day 7. BMMSCs markedly attenuated allograft rejection, with fewer infiltrating cells and apoptotic bodies, and reduced crypt epithelial injury and mucosal ulceration until day 7. By day 10, however, the improvement was attenuated, likely because of the reduction in numbers of BMMSCs and their diminished effects in vivo. Ultrastructural examination showed that severe damage to microvilli and tight junctions were repaired on day 7 in the isogeneic control group, coupled with recovery from IRI, but damage was not alleviated in the allogeneically transplanted animals, in agreement with previous results [26]. BMMSCs protected the ultrastructure from injury after allogeneic SBTx until day 7, but these effects were attenuated on day 10.

NK cells mediate the non-MHC–restricted killing of virally infected and neoplastic cells and are involved in regulating the immune responses. Human NK cells mediate the rejection of both allogeneic bone marrow and xenogeneic solid organ grafts [30], [31]. We observed significantly increased NK cell activity in the Allo control group and that BMMSCs induced NK cell activity, which consequently alleviated acute rejection after allotransplantation.

Pro- and anti-inflammatory cytokines play an important role in transplantation immunology. Serum concentrations of IL-2 increase during acute rejection and are highly correlated with the severity of rejection [32]. In contrast, high concentrations of IL-10 are associated with non-rejection of solid allografts and the induction of transplantation tolerance [33]. IL-10 has been reported to inhibit Th1 immune responses by blocking the synthesis of IL-12 in dendritic cells and inducing Th2 responses [34]. Serum IL-2 and IL-10 concentrations and the IL-2/IL-10 ratio have been found to correlate with rejection and tolerance in SBTx [35]. We found that BMMSCs significantly decreased IL-2 and increased IL-10 concentrations in both serum and graft when compared with allogeneically transplanted animals, with the highest IL-2/IL-10 ratio observed on day 7. BMMSC treatment also increased the concentration of TGF-β, an anti-inflammatory cytokine secreted by MSCs to regulate immune responses, within 10 days. The pro-inflammatory cytokine IFN-γ, which is associated with Th1, was significantly induced in the BMMSC group. TNF-α upregulation after transplantation has been associated with immunoregulatory processes, apoptosis induction and T-cell proliferation [36], [37]. TNF-α concentrations were shown to correlate positively with the grade of acute rejection in human and experimental solid organ transplantation, including SBTx [38], [39], [40], [41]. We also found that TNF-α concentrations increased in the serum and graft of the allogeneic control group, but that these concentrations were reduced by BMMSC treatment, accompanied by a reduction in apoptosis in the intestinal mucosa.

The switch of the Th17/Treg axis plays an important role in regulating immune responses and autoimmune disease [42], [43]. Regulation of the Treg/Th17 balance involved in inflammation and autoimmune diseases may play an important role in promoting or suppressing immune rejection. Th17/Treg regulatory functions were assessed from several aspects, including their related cytokine secretion. Our study demonstrates that there was significantly increased expression of Treg-associated cytokines (IL-10 and TGF-β) in the BMMSC group, accompanied by remarkably decreased expression of Th17-related cytokines (IL-17, IL-6, IL-23, IFN-γ, TNF-α) when compared to that in the Allogeneic control group.

Tregs (CD4^+^CD25^+^Foxp3^+^ T cells) are a subset of T cells that play a critical role in inducing and maintaining immunologic tolerance to both self and foreign antigens by suppressing aggressive T cell responses [44]. The suppressive effects of Treg cells are partly related to cytokines such as TGF-β and IL-10 [45], and Treg cells were shown to prolong allograft survival [46], [47], [48]. We found that BMMSC treatment increased the populations of CD4^+^CD25^+^Foxp3^+^ T cells, suggesting that this increase can protect allografts from ACR. In the Tregs-Depleted group, there were poorer histopathological results and apoptosis compared to the BMMSC group, but these aspects were improved compared to the Allogeneic control group. Serum IL-10 and TGF-β concentrations were decreased, while serum IL-2, IL-6, IL-17, IL-23, TNF-α, and IFN-γ increased by varying degrees compared to the BMMSC group. On the contrary, when compared to the Allogeneic control group, serum IL-10 was increased while IL-2, IL-6, IL-17, IL-23, TNF-α, and IFN-γ were decreased in the Tregs-Depleted group. Therefore, we conclude that inducing Treg production is just one of the mechanism by which BMMSCs display their immunosuppressive effect.

In summary, we found that infusion of BMMSCs suppressed acute rejection in SBTx, and that the immunoregulatory effect of these cells is due to the balance of Th1/Th2, Th17/Treg, and their related cytokines (IL-10, TGF-β, IL-2, IL-6, IL-17, IL-23, TNF-α, IFN-γ) and NK cell activity, as well as Treg expansion. Although the suppressive effect decreased over time, due to reduction of live BMMSCs, these findings suggest a new strategy for suppressing ACR in SBTx.

## Supporting Information

S1 Table **Serum cytokines concentrations in each group.**
(DOCX)

S2 Table **Graft cytokines concentrations in each group.**
(DOCX)
