# Correction: Reduction of Acute Rejection by Bone Marrow Mesenchymal Stem Cells during Rat Small Bowel Transplantation

**DOI:** 10.1371/journal.pone.0118717

**Published:** 2015-03-05

**Authors:** The PLOS ONE Staff

The seventh author’s name is spelled incorrectly. The correct name is: Chong Dong.
