# Supplementary material for: Reduction of Acute Rejection by Bone Marrow Mesenchymal Stem Cells during Rat Small Bowel Transplantation
Source: PLoS One. 2014 Dec 15;9(12):e114528. doi: 10.1371/journal.pone.0114528 (PMC4266507; doi:10.1371/journal.pone.0114528)
Supplement: S1 Table — Serum cytokines concentrations in each group. (DOCX) [file pone.0114528.s001.docx]

Table S1: Serum cytokines concentrations in each group.

| Group | Time (day) | Serum IL-10 concentrations (pg/ml) | | | | |
| --- | --- | --- | --- | --- | --- | --- |
|  |  | Sample 1 | Sample 2 | Sample 3 | Sample 4 | Sample 5 |
| NSBT |  | 21.605 | 16.435 | 17.703 | 18.267 | 20.479 |
| Iso | 1 | 17.434 | 21.235 | 19.262 | 18.228 | 23.463 |
|  | 5 | 23.686 | 20.874 | 21.942 | 18.883 | 25.497 |
|  | 7 | 18.415 | 20.228 | 17.394 | 23.624 | 21.633 |
|  | 10 | 20.238 | 17.947 | 15.336 | 19.066 | 21.492 |
| Allo | 1 | 21.547 | 18.675 | 20.626 | 17.911 | 23.059 |
|  | 5 | 21.811 | 24.793 | 25.605 | 26.768 | 22.172 |
|  | 7 | 27.946 | 25.885 | 24.306 | 28.606 | 30.846 |
|  | 10 | 24.178 | 27.563 | 28.049 | 30.292 | 32.064 |
| BMMSCs | 1 | 24.685 | 25.874 | 22.942 | 19.883 | 20.323 |
|  | 5 | 27.833 | 29.468 | 31.293 | 34.176 | 28.476 |
|  | 7 | 35.246 | 39.633 | 42.947 | 36.336 | 40.264 |
|  | 10 | 45.629 | 42.176 | 37.836 | 40.468 | 48.724 |
| Group | Time (day) | Serum TGF-β concentrations (pg/ml) | | | | |
|  |  | Sample 1 | Sample 2 | Sample 3 | Sample 4 | Sample 5 |
| NSBT |  | 37.432 | 40.857 | 36.364 | 41.326 | 34.593 |
| Iso | 1 | 41.556 | 37.603 | 39.407 | 42.857 | 43.826 |
|  | 5 | 46.857 | 38.823 | 48.112 | 43.014 | 44.849 |
|  | 7 | 40.675 | 42.011 | 37.612 | 34.688 | 43.213 |
|  | 10 | 35.152 | 41.232 | 36.776 | 42.523 | 38.307 |
| Allo | 1 | 40.323 | 47.528 | 44.746 | 39.143 | 45.103 |
|  | 5 | 51.264 | 44.208 | 47.617 | 50.247 | 52.352 |
|  | 7 | 57.574 | 55.152 | 49.321 | 47.836 | 54.486 |
|  | 10 | 56.642 | 59.562 | 53.192 | 54.788 | 50.782 |
| BMMSCs | 1 | 41.958 | 44.219 | 38.136 | 43.688 | 37.459 |
|  | 5 | 51.137 | 55.329 | 58.691 | 56.682 | 48.705 |
|  | 7 | 64.285 | 68.322 | 55.573 | 59.194 | 71.536 |
|  | 10 | 65.925 | 72.495 | 68.837 | 76.156 | 70.184 |
| Group | Time (day) | Serum IL-2 concentrations (pg/ml) | | | | |
|  |  | Sample 1 | Sample 2 | Sample 3 | Sample 4 | Sample 5 |
| NSBT |  | 31.331 | 35.302 | 29.632 | 34.532 | 32.529 |
| Iso | 1 | 41.047 | 36.855 | 35.141 | 38.596 | 33.04 |
|  | 5 | 38.105 | 42.287 | 45.428 | 37.556 | 46.458 |
|  | 7 | 37.447 | 31.658 | 30.478 | 36.734 | 34.372 |
|  | 10 | 28.256 | 34.728 | 29.384 | 34.293 | 35.385 |
| Allo | 1 | 43.583 | 40.641 | 37.342 | 35.173 | 38.948 |
|  | 5 | 57.353 | 53.063 | 60.916 | 54.634 | 48.932 |
|  | 7 | 65.463 | 75.246 | 62.612 | 69.713 | 67.288 |
|  | 10 | 93.501 | 86.815 | 91.804 | 85.426 | 81.501 |
| BMMSCs | 1 | 34.842 | 41.854 | 38.778 | 37.345 | 35.395 |
|  | 5 | 45.516 | 47.86 | 38.644 | 44.219 | 40.462 |
|  | 7 | 53.298 | 51.378 | 47.121 | 44.393 | 54.817 |
|  | 10 | 68.303 | 72.435 | 77.685 | 65.835 | 70.683 |
| Group | Time (day) | Serum IL-6 concentrations (pg/ml) | | | | |
|  |  | Sample 1 | Sample 2 | Sample 3 | Sample 4 | Sample 5 |
| NSBT |  | 93.563 | 87.616 | 81.477 | 101.263 | 96.066 |
| Iso | 1 | 105.899 | 90.931 | 97.899 | 103.931 | 87.673 |
|  | 5 | 114.743 | 102.683 | 107.726 | 98.641 | 120.466 |
|  | 7 | 91.478 | 103.311 | 95.437 | 106.297 | 88.099 |
|  | 10 | 85.345 | 89.412 | 103.376 | 92.369 | 98.852 |
| Allo | 1 | 102.453 | 93.528 | 105.746 | 99.143 | 115.103 |
|  | 5 | 133.834 | 124.208 | 137.617 | 142.247 | 112.352 |
|  | 7 | 150.395 | 155.152 | 139.321 | 167.836 | 154.486 |
|  | 10 | 180.439 | 167.562 | 195.192 | 174.788 | 187.782 |
| BMMSCs | 1 | 108.515 | 104.481 | 94.546 | 87.461 | 97.661 |
|  | 5 | 65.852 | 57.853 | 71.861 | 73.866 | 61.293 |
|  | 7 | 51.669 | 64.595 | 60.738 | 48.628 | 55.094 |
|  | 10 | 86.947 | 83.673 | 70.864 | 76.778 | 81.399 |
| Group | Time (day) | Serum IL-17 concentrations (pg/ml) | | | | |
|  |  | Sample 1 | Sample 2 | Sample 3 | Sample 4 | Sample 5 |
| NSBT |  | 34.211 | 41.112 | 39.242 | 36.137 | 40.797 |
| Iso | 1 | 34.607 | 31.741 | 41.549 | 37.797 | 43.873 |
|  | 5 | 46.274 | 52.181 | 55.244 | 48.177 | 41.741 |
|  | 7 | 40.476 | 36.582 | 35.43 | 42.553 | 45.299 |
|  | 10 | 34.852 | 32.399 | 38.676 | 41.425 | 30.791 |
| Allo | 1 | 38.286 | 50.764 | 45.914 | 41.832 | 46.688 |
|  | 5 | 53.468 | 56.513 | 61.685 | 58.569 | 65.828 |
|  | 7 | 91.715 | 85.704 | 83.75 | 95.695 | 77.616 |
|  | 10 | 108.75 | 103.682 | 115.722 | 126.592 | 118.793 |
| BMMSCs | 1 | 32.662 | 38.171 | 27.682 | 34.173 | 30.695 |
|  | 5 | 21.263 | 24.236 | 25.24 | 30.225 | 28.887 |
|  | 7 | 20.851 | 23.905 | 24.829 | 27.904 | 18.507 |
|  | 10 | 42.381 | 35.389 | 38.408 | 44.425 | 47.814 |
| Group | Time (day) | Serum IL-23 concentrations (pg/ml) | | | | |
|  |  | Sample 1 | Sample 2 | Sample 3 | Sample 4 | Sample 5 |
| NSBT |  | 47.502 | 45.024 | 51.355 | 54.953 | 50.392 |
| Iso | 1 | 52.457 | 61.788 | 48.573 | 55.825 | 45.649 |
|  | 5 | 61.401 | 52.867 | 65.023 | 58.87 | 69.274 |
|  | 7 | 54.745 | 60.585 | 63.738 | 57.583 | 45.184 |
|  | 10 | 47.599 | 53.261 | 58.585 | 51.627 | 62.831 |
| Allo | 1 | 54.537 | 60.625 | 56.519 | 45.639 | 61.625 |
|  | 5 | 81.157 | 74.429 | 77.171 | 84.412 | 92.423 |
|  | 7 | 90.961 | 88.961 | 83.956 | 103.942 | 97.245 |
|  | 10 | 101.431 | 112.54 | 108.435 | 117.548 | 123.438 |
| BMMSCs | 1 | 46.806 | 53.773 | 41.811 | 44.786 | 50.035 |
|  | 5 | 32.609 | 28.645 | 44.603 | 37.673 | 38.176 |
|  | 7 | 24.801 | 35.104 | 25.797 | 32.067 | 27.133 |
|  | 10 | 47.478 | 35.325 | 38.354 | 44.362 | 53.277 |
| Group | Time (day) | Serum TNF-α concentrations (pg/ml) | | | | |
|  |  | Sample 1 | Sample 2 | Sample 3 | Sample 4 | Sample 5 |
| NSBT |  | 38.608 | 41.492 | 38.497 | 39.562 | 43.538 |
| Iso | 1 | 41.908 | 45.659 | 48.165 | 42.256 | 39.062 |
|  | 5 | 54.951 | 52.993 | 49.509 | 50.613 | 57.353 |
|  | 7 | 43.389 | 47.312 | 42.769 | 40.303 | 48.819 |
|  | 10 | 42.945 | 38.684 | 40.471 | 35.911 | 45.934 |
| Allo | 1 | 48.289 | 45.711 | 42.972 | 46.688 | 41.506 |
|  | 5 | 64.415 | 56.711 | 59.116 | 69.228 | 63.303 |
|  | 7 | 77.913 | 79.529 | 71.715 | 67.285 | 74.562 |
|  | 10 | 89.985 | 85.128 | 92.626 | 98.811 | 94.125 |
| BMMSCs | 1 | 45.796 | 44.527 | 40.494 | 48.788 | 42.122 |
|  | 5 | 56.904 | 60.642 | 51.658 | 53.405 | 58.067 |
|  | 7 | 63.117 | 60.217 | 54.595 | 57.461 | 62.203 |
|  | 10 | 76.493 | 81.285 | 73.107 | 69.694 | 78.463 |
| Group | Time (day) | Serum IFN-γ concentrations (pg/ml) | | | | |
|  |  | Sample 1 | Sample 2 | Sample 3 | Sample 4 | Sample 5 |
| NSBT |  | 59.015 | 72.835 | 64.162 | 73.538 | 68.727 |
| Iso | 1 | 72.252 | 68.662 | 78.234 | 75.923 | 63.602 |
|  | 5 | 88.274 | 94.288 | 98.307 | 105.868 | 83.484 |
|  | 7 | 80.323 | 83.559 | 71.217 | 73.533 | 68.176 |
|  | 10 | 66.252 | 68.291 | 73.539 | 62.365 | 75.975 |
| Allo | 1 | 78.221 | 74.357 | 69.354 | 83.032 | 87.647 |
|  | 5 | 155.465 | 142.559 | 132.182 | 138.918 | 124.343 |
|  | 7 | 152.694 | 164.383 | 176.305 | 173.543 | 186.042 |
|  | 10 | 206.365 | 194.176 | 186.047 | 216.177 | 223.067 |
| BMMSCs | 1 | 70.604 | 68.589 | 75.816 | 78.308 | 65.759 |
|  | 5 | 92.515 | 96.716 | 109.188 | 111.181 | 108.251 |
|  | 7 | 126.308 | 117.834 | 111.591 | 103.294 | 101.748 |
|  | 10 | 155.125 | 142.294 | 169.819 | 146.166 | 172.83 |
