# Supplementary material for: Reduction of Acute Rejection by Bone Marrow Mesenchymal Stem Cells during Rat Small Bowel Transplantation
Source: PLoS One. 2014 Dec 15;9(12):e114528. doi: 10.1371/journal.pone.0114528 (PMC4266507; doi:10.1371/journal.pone.0114528)
Supplement: S2 Table — Graft cytokines concentrations in each group. (DOCX) [file pone.0114528.s002.docx]

Table S2: Graft cytokines concentrations in each group.

| Group | Time (day) | Graft IL-10 concentrations (pg/mg protein) | | | | |
| --- | --- | --- | --- | --- | --- | --- |
|  |  | Sample 1 | Sample 2 | Sample 3 | Sample 4 | Sample 5 |
| NSBT |  | 36.539 | 33.192 | 40.208 | 37.617 | 38.247 |
| Iso | 1 | 45.812 | 42.786 | 38.248 | 47.285 | 40.768 |
|  | 5 | 49.045 | 53.105 | 58.101 | 51.707 | 46.771 |
|  | 7 | 54.513 | 48.836 | 43.303 | 47.811 | 51.203 |
|  | 10 | 39.731 | 42.608 | 44.279 | 45.303 | 36.783 |
| Allo | 1 | 46.577 | 38.293 | 42.121 | 45.912 | 49.848 |
|  | 5 | 47.265 | 53.476 | 57.126 | 45.913 | 50.533 |
|  | 7 | 52.185 | 49.605 | 60.553 | 58.557 | 46.404 |
|  | 10 | 52.238 | 55.724 | 45.893 | 59.113 | 43.753 |
| BMMSCs | 1 | 40.512 | 52.046 | 43.076 | 46.685 | 50.604 |
|  | 5 | 54.615 | 67.546 | 61.311 | 57.557 | 64.749 |
|  | 7 | 77.932 | 63.307 | 67.739 | 69.108 | 73.627 |
|  | 10 | 75.645 | 79.907 | 68.644 | 82.728 | 87.433 |
| Group | Time (day) | Graft TGF-β concentrations (pg/mg protein) | | | | |
|  |  | Sample 1 | Sample 2 | Sample 3 | Sample 4 | Sample 5 |
| NSBT |  | 83.737 | 71.546 | 79.708 | 76.629 | 74.665 |
| Iso | 1 | 81.219 | 87.285 | 73.593 | 91.069 | 83.398 |
|  | 5 | 91.957 | 96.553 | 83.147 | 109.54 | 102.568 |
|  | 7 | 95.091 | 89.058 | 90.534 | 98.955 | 83.275 |
|  | 10 | 88.366 | 84.242 | 77.682 | 73.254 | 80.868 |
| Allo | 1 | 84.283 | 89.367 | 77.936 | 95.035 | 92.572 |
|  | 5 | 93.834 | 99.413 | 115.204 | 87.631 | 108.304 |
|  | 7 | 111.338 | 105.788 | 116.477 | 121.612 | 98.741 |
|  | 10 | 118.843 | 103.498 | 95.577 | 112.103 | 106.517 |
| BMMSCs | 1 | 81.141 | 84.042 | 75.23515 | 96.402 | 92.695 |
|  | 5 | 121.135 | 112.578 | 106.429 | 117.289 | 125.936 |
|  | 7 | 133.089 | 145.725 | 136.128 | 156.495 | 127.597 |
|  | 10 | 164.511 | 147.831 | 172.414 | 140.887 | 135.721 |
| Group | Time (day) | Graft IL-2 concentrations (pg/mg protein) | | | | |
|  |  | Sample 1 | Sample 2 | Sample 3 | Sample 4 | Sample 5 |
| NSBT |  | 44.288 | 41.836 | 46.615 | 40.706 | 49.604 |
| Iso | 1 | 52.494 | 49.196 | 46.213 | 53.594 | 56.195 |
|  | 5 | 55.91 | 53.445 | 48.626 | 60.248 | 50.451 |
|  | 7 | 41.744 | 47.149 | 42.273 | 50.633 | 45.503 |
|  | 10 | 43.502 | 39.907 | 46.374 | 50.298 | 48.716 |
| Allo | 1 | 57.258 | 55.278 | 49.504 | 60.271 | 62.386 |
|  | 5 | 79.706 | 70.609 | 74.581 | 83.784 | 67.341 |
|  | 7 | 95.139 | 87.584 | 91.946 | 92.802 | 101.643 |
|  | 10 | 124.734 | 112.155 | 108.657 | 118.778 | 121.946 |
| BMMSCs | 1 | 60.688 | 54.508 | 59.042 | 48.096 | 56.765 |
|  | 5 | 56.806 | 64.817 | 66.927 | 60.842 | 53.269 |
|  | 7 | 73.052 | 66.308 | 62.478 | 68.394 | 75.401 |
|  | 10 | 80.938 | 92.372 | 95.417 | 101.176 | 84.178 |
| Group | Time (day) | Graft IL-6 concentrations (pg/mg protein) | | | | |
|  |  | Sample 1 | Sample 2 | Sample 3 | Sample 4 | Sample 5 |
| NSBT |  | 82.273 | 80.633 | 75.503 | 73.052 | 66.308 |
| Iso | 1 | 72.494 | 85.910 | 81.744 | 73.502 | 77.258 |
|  | 5 | 89.196 | 93.445 | 97.149 | 79.907 | 85.278 |
|  | 7 | 81.213 | 75.626 | 72.288 | 91.374 | 87.504 |
|  | 10 | 72.594 | 92.248 | 78.836 | 75.298 | 82.271 |
| Allo | 1 | 86.195 | 90.451 | 93.615 | 80.716 | 75.386 |
|  | 5 | 104.688 | 96.806 | 115.706 | 108.938 | 124.734 |
|  | 7 | 120.508 | 133.817 | 126.604 | 138.372 | 112.155 |
|  | 10 | 155.042 | 164.927 | 142.478 | 135.417 | 128.657 |
| BMMSCs | 1 | 79.096 | 70.842 | 68.394 | 85.176 | 74.778 |
|  | 5 | 56.765 | 63.269 | 65.401 | 74.178 | 78.946 |
|  | 7 | 60.706 | 72.139 | 64.581 | 53.946 | 57.341 |
|  | 10 | 75.609 | 87.584 | 83.784 | 92.802 | 98.643 |
| Group | Time (day) | Graft IL-17 concentrations (pg/mg protein) | | | | |
|  |  | Sample 1 | Sample 2 | Sample 3 | Sample 4 | Sample 5 |
| NSBT |  | 40.932 | 43.307 | 45.739 | 49.108 | 54.627 |
| Iso | 1 | 43.645 | 45.812 | 49.045 | 54.513 | 57.731 |
|  | 5 | 59.907 | 62.786 | 51.105 | 57.836 | 66.608 |
|  | 7 | 61.644 | 50.248 | 58.101 | 43.303 | 44.279 |
|  | 10 | 58.728 | 40.285 | 51.707 | 47.811 | 45.303 |
| Allo | 1 | 57.433 | 63.768 | 48.771 | 51.203 | 54.783 |
|  | 5 | 76.265 | 82.185 | 63.238 | 70.512 | 84.615 |
|  | 7 | 93.476 | 80.605 | 76.724 | 101.046 | 87.546 |
|  | 10 | 108.126 | 99.553 | 85.893 | 93.076 | 117.311 |
| BMMSCs | 1 | 42.913 | 45.557 | 36.113 | 51.685 | 54.557 |
|  | 5 | 30.533 | 43.404 | 40.753 | 27.604 | 36.749 |
|  | 7 | 22.577 | 36.539 | 37.617 | 33.192 | 28.848 |
|  | 10 | 52.293 | 57.121 | 48.912 | 43.208 | 65.247 |
| Group | Time (day) | Graft IL-23 concentrations (pg/mg protein) | | | | |
|  |  | Sample 1 | Sample 2 | Sample 3 | Sample 4 | Sample 5 |
| NSBT |  | 33.737 | 41.546 | 39.708 | 46.629 | 44.665 |
| Iso | 1 | 41.219 | 38.285 | 43.593 | 49.069 | 52.398 |
|  | 5 | 54.957 | 46.553 | 43.147 | 49.540 | 58.568 |
|  | 7 | 45.091 | 39.058 | 40.534 | 34.955 | 51.275 |
|  | 10 | 48.366 | 52.242 | 37.682 | 34.254 | 40.868 |
| Allo | 1 | 54.283 | 46.367 | 39.936 | 45.035 | 50.572 |
|  | 5 | 61.834 | 67.413 | 73.204 | 75.631 | 56.304 |
|  | 7 | 71.338 | 63.788 | 76.477 | 84.612 | 68.741 |
|  | 10 | 78.843 | 103.498 | 95.577 | 92.103 | 83.517 |
| BMMSCs | 1 | 28.141 | 33.042 | 35.232 | 43.402 | 42.695 |
|  | 5 | 24.135 | 32.578 | 36.429 | 27.289 | 25.936 |
|  | 7 | 33.089 | 42.725 | 36.128 | 28.495 | 38.597 |
|  | 10 | 54.511 | 47.831 | 52.414 | 43.887 | 36.721 |
| Group | Time (day) | Graft TNF-α concentrations (pg/mg protein) | | | | |
|  |  | Sample 1 | Sample 2 | Sample 3 | Sample 4 | Sample 5 |
| NSBT |  | 11.248 | 15.653 | 13.932 | 9.385 | 12.567 |
| Iso | 1 | 12.917 | 17.124 | 14.591 | 10.546 | 12.507 |
|  | 5 | 16.755 | 19.404 | 17.848 | 23.263 | 25.603 |
|  | 7 | 20.185 | 23.236 | 17.413 | 15.306 | 18.312 |
|  | 10 | 15.188 | 10.328 | 17.525 | 12.372 | 16.367 |
| Allo | 1 | 18.308 | 15.653 | 20.821 | 12.419 | 14.194 |
|  | 5 | 25.283 | 28.407 | 21.807 | 22.902 | 25.488 |
|  | 7 | 32.261 | 37.153 | 29.941 | 34.959 | 35.631 |
|  | 10 | 50.263 | 42.653 | 49.464 | 44.531 | 47.632 |
| BMMSCs | 1 | 19.944 | 15.288 | 16.728 | 13.066 | 14.353 |
|  | 5 | 15.659 | 16.485 | 21.355 | 25.967 | 18.325 |
|  | 7 | 25.807 | 22.703 | 23.151 | 26.548 | 29.805 |
|  | 10 | 31.388 | 37.995 | 40.388 | 42.562 | 35.854 |
| Group | Time (day) | Graft IFN-γ concentrations (pg/mg protein) | | | | |
|  |  | Sample 1 | Sample 2 | Sample 3 | Sample 4 | Sample 5 |
| NSBT |  | 101.248 | 115.653 | 123.932 | 109.385 | 92.567 |
| Iso | 1 | 124.917 | 107.124 | 134.591 | 142.546 | 112.507 |
|  | 5 | 146.755 | 139.404 | 157.848 | 165.263 | 125.603 |
|  | 7 | 120.185 | 133.236 | 117.413 | 125.306 | 108.312 |
|  | 10 | 115.188 | 102.328 | 122.525 | 132.372 | 96.367 |
| Allo | 1 | 138.308 | 145.653 | 124.821 | 112.419 | 154.194 |
|  | 5 | 155.283 | 188.407 | 201.807 | 192.902 | 175.488 |
|  | 7 | 212.261 | 237.153 | 189.941 | 194.959 | 175.631 |
|  | 10 | 230.263 | 242.653 | 197.464 | 264.531 | 217.632 |
| BMMSCs | 1 | 109.944 | 115.288 | 126.728 | 93.066 | 134.353 |
|  | 5 | 115.659 | 126.485 | 151.355 | 145.967 | 138.325 |
|  | 7 | 118.807 | 134.703 | 173.151 | 156.548 | 149.805 |
|  | 10 | 171.388 | 187.995 | 200.388 | 192.562 | 155.854 |
